# Dimerization of sortilin regulates its trafficking to extracellular vesicles

**DOI:** 10.1074/jbc.RA117.000732

**Published:** 2018-01-30

**Authors:** Shinsuke Itoh, Ken Mizuno, Masanori Aikawa, Elena Aikawa

**Affiliations:** From the ‡ Center for Interdisciplinary Cardiovascular Sciences and; ¶ Center for Excellence in Vascular Biology, Cardiovascular Division, Brigham and Women's Hospital, Harvard Medical School, Boston, Massachusetts 02115 and; § Tokyo New Drug Research Laboratories, Kowa Company, Ltd., Tokyo 189-0022, Japan

**Keywords:** dimerization, extracellular vesicles, trafficking, calcification, sorting, intermolecular disulfide bond, sortilin

## Abstract

Extracellular vesicles (EVs) play a critical role in intercellular communication by transferring microRNAs, lipids, and proteins to neighboring cells. Sortilin, a sorting receptor that directs target proteins to the secretory or endocytic compartments of cells, is found in both EVs and cells. In many human diseases, including cancer and cardiovascular disorders, sortilin expression levels are atypically high. To elucidate the relationship between cardiovascular disease, particularly vascular calcification, and sortilin expression levels, we explored the trafficking of sortilin in both the intracellular and extracellular milieu. We previously demonstrated that sortilin promotes vascular calcification via its trafficking of tissue-nonspecific alkaline phosphatase to EVs. Although recent reports have noted that sortilin is regulated by multiple post-translational modifications, the precise mechanisms of sortilin trafficking still need to be determined. Here, we show that sortilin forms homodimers with an intermolecular disulfide bond at the cysteine 783 (Cys^783^) residue, and because Cys^783^ can be palmitoylated, it could be shared via palmitoylation and an intermolecular disulfide bond. Formation of this intermolecular disulfide bond leads to trafficking of sortilin to EVs by preventing palmitoylation, which further promotes sortilin trafficking to the Golgi apparatus. Moreover, we found that sortilin-derived propeptide decreased sortilin homodimers within EVs. In conclusion, sortilin is transported to EVs via the formation of homodimers with an intermolecular disulfide bond, which is endogenously regulated by its own propeptide. Therefore, we propose that inhibiting dimerization of sortilin acts as a new therapeutic strategy for the treatment of EV-associated diseases, including vascular calcification and cancer.

## Introduction

Intercellular communication, an essential hallmark of multicellular organisms, can be mediated through direct cell–cell contact or the transfer of secreted molecules (1). In the last two decades, a new mechanism for intercellular communication has emerged that involves intercellular transfer of extracellular vesicles (EVs),^3^ such as exosomes, which have the ability to transfer their cellular content to neighboring cells and to modify the cellular microenvironment (2, 3). The role of EVs is likely to be dictated by the vesicle cargo, typically composed of microRNAs, RNAs, lipids, and/or proteins. However, the function of some of these proteins in EVs and how they affect various diseases need further exploration.

Sortilin, which is ubiquitously expressed and essential for proper function of many tissues and cell types, is a sorting receptor that directs target proteins, including growth factors, signaling receptors, and enzymes, to their destined location in the secretory or endocytic compartments of cells (4). Sortilin has conversely also emerged as a major cause of malignancies in a range of diseases (5), including cancer (6–9), type 2 diabetes mellitus (10), hypercholesterolemia (11–13), atherosclerosis (14, 15), and neurodegenerative disorders (16, 17), such as Alzheimer's disease (18, 19). The atypical increase in intracellular trafficking by sortilin and its subsequent lysosomal degradation (16) or secretion (11, 15) have been linked to the pathogenesis of the aforementioned diseases. In addition, recent studies have shown that sortilin could convey causative molecules of diseases to the extracellular space via EVs: 1) previous studies have shown that sortilin transports tyrosine kinases to neighboring cells through exosome transfer, promoting tumorigenesis via activation of angiogenesis (7), and 2) our recent research has demonstrated that sortilin promotes vascular calcification via its trafficking and loading of tissue-nonspecific alkaline phosphatase into EVs (20).

Therefore, our major objective is to understand the process that facilitates the transport of sortilin into EVs. Addressing this question could help to develop new therapeutic approaches for EV-associated diseases. Although multiple post-translational modifications, including phosphorylation (20), ubiquitination (21, 22), and palmitoylation (23), regulate functions of sortilin, the mechanisms controlling sortilin trafficking have yet to be fully understood. Because the trafficking of receptors, such as G-protein–coupled receptors (24) and type I transmembrane proteins (25, 26), can be regulated by dimerization, we hypothesized that dimerization is a major regulator of sortilin trafficking to EVs. Here, we provide the first evidence that sortilin forms homodimers, thereby facilitating its trafficking to EVs. Specifically, we showed that 1) sortilin forms homodimers with an intermolecular disulfide bond at Cys^783^, 2) mutation of Cys^783^ abolishes transport of dimerized sortilin to EVs, and 3) inhibition of palmitoylation at Cys^783^ increases sortilin homodimers. Our results indicate that Cys^783^ acts as a biological switch to destine the trafficking of sortilin: the intermolecular disulfide bond promotes the trafficking of sortilin to EVs, and palmitoylation advances it further to the Golgi apparatus (23). Moreover, we found that sortilin-derived propeptide decreases sortilin homodimers in EVs. We therefore propose a new mechanism for regulating trafficking of sortilin through its dimerization with an intermolecular disulfide bond, which is regulated via ligand binding in the extracellular domain.

## Results

### Sortilin forms homodimers on the cell surface

**Figure 1.**
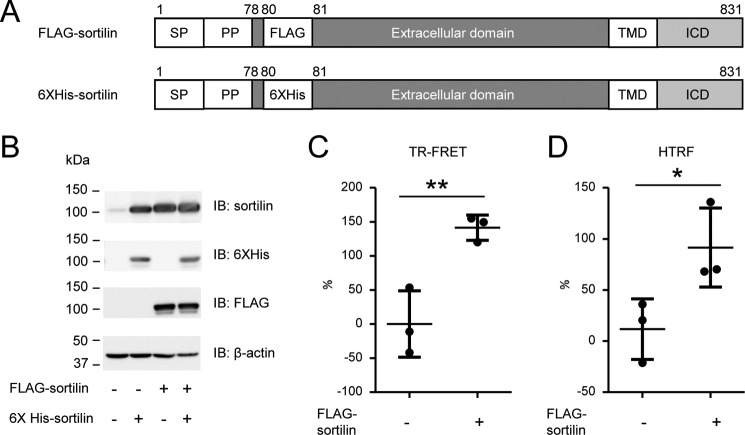
**Sortilin forms homodimers on the cell surface of HEK293 cells.** *A*, schematic of FLAG-sortilin and His_6_-sortilin. FLAG tag and His_6_ tag were inserted following propeptide and 3 amino acids (Ser^78^-Ala^79^-Pro^80^) in sortilin. *SP*, signal peptide; *PP*, propeptide. *B*, overexpression of FLAG-sortilin and His_6_-sortilin in HEK293 cells was validated by Western blotting. *C* and *D*, detection of binding of FLAG-sortilin and His_6_-sortilin on the cell surface of HEK293 in TR-FRET assay (*C*) and HTRF assay (*D*). Change of FRET signal by expression of His_6_-sortilin is indicated by percent change (mean ± S.D., three independent experiments). *Error bars* represent S.D. *, *p* < 0.05; **, *p* < 0.01 by *t* test. *IB*, immunoblotting.

We performed a time-resolved fluorescence energy transfer (TR-FRET) assay to detect sortilin homodimerization. Expression vectors of FLAG-sortilin and His_6_-sortilin were constructed for the TR-FRET assay (Fig. 1*A*). FLAG tag and His_6_ tag were placed following propeptide and 3 amino acids (Ser-Ala-Pro) to detect the extracellular domains of FLAG-sortilin and His_6_-sortilin after propeptide cleavage (Fig. 1*A*). Both FLAG-sortilin and His_6_-sortilin were overexpressed in HEK293 cells. Protein expression of FLAG-sortilin and His_6_-sortilin was validated by Western blotting (Fig. 1*B*). This coexpression increased the FRET signal when compared with HEK293 cells overexpressing only His_6_-sortilin (Fig. 1*C*), indicating that sortilin forms homodimers on the cell surface. Also, an increased FRET signal was detected in homogenous TR-FRET (HTRF), a result that aligns with previous reports (27) (Fig. 1*D*). These results indicate that our FRET assay is effective in screening for molecules involved in sortilin dimerization.

### Sortilin forms homodimers in the extracellular and intracellular domains with intermolecular disulfide bonds

**Figure 2.**
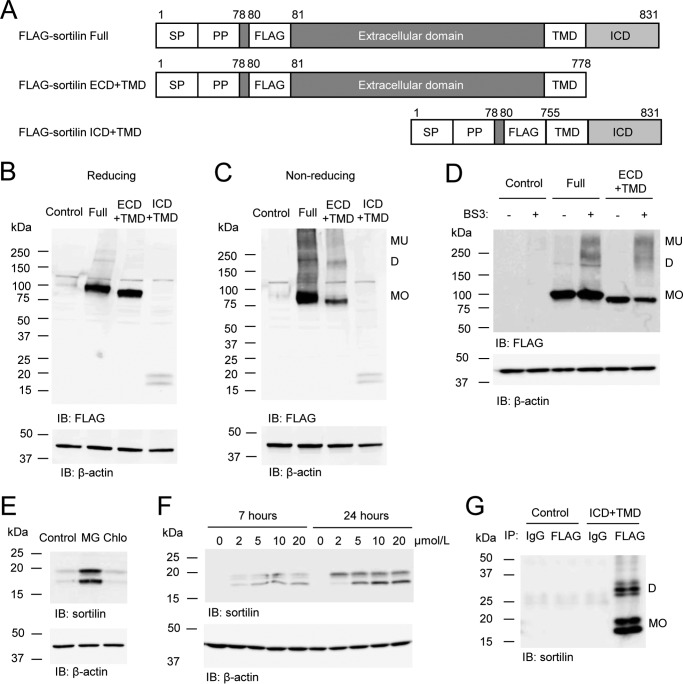
**Sortilin forms homodimers in the extracellular and intracellular domains with intermolecular disulfide bonds in HEK293 cells.** *A*, schematic of FLAG-sortilin Full, ECD+TMD, and ICD+TMD. *SP*, signal peptide; *PP*, propeptide. *B* and *C*, protein expression of FLAG-sortilin Full, ECD+TMD, and ICD+TMD was validated in reducing (*B*) and non-reducing (*C*) Western blotting using anti-FLAG antibody. FLAG-sortilin Full and ECD+TMD form homodimers and multimers. Empty vector was used as a control. *D*, HEK293 cells transiently overexpressing FLAG-sortilin Full or ECD+TMD were treated with a cross-linker, BS3, and the cell lysates were used for reducing Western blotting with anti-FLAG antibody, showing dimerization of sortilin Full and ECD+TMD (*n* = 3). *E*, HEK293 cells stably overexpressing FLAG-sortilin ICD+TMD (FLAG-sortilin ICD+TMD HEK293 cells) were incubated with DMSO (*Control*), 20 μmol/liter MG-132 (*MG*) or 10 μmol/liter chloroquine (*Chlo*) for 7 h, and then reducing Western blotting was performed using anti-sortilin antibody. MG-132 increased the protein expression of FLAG-sortilin ICD+TMD, but chloroquine did not (*n* = 3). *F*, FLAG-sortilin ICD+TMD HEK293 cells were incubated with DMSO or MG-132 (2–20 μmol/liter) for 7 or 24 h. MG-132 increased FLAG-sortilin ICD+TMD in a time- and concentration-dependent manner (*n* = 3). *G*, following 16-h incubation of HEK293 cells (*Control*) or FLAG-sortilin ICD+TMD HEK293 cells (*ICD*+*TMD*) with MG-132 (5 μmol/liter) and immunoprecipitation with anti-FLAG antibody, non-reducing Western blotting showed dimerization of sortilin ICD+TMD using anti-sortilin antibody (*n* = 3). Monomers, homodimers, and multimers are abbreviated as *MO*, *D*, and *MU*, respectively. *IB*, immunoblotting.

To investigate whether the extracellular domain (ECD) or intracellular domain (ICD) is responsible for the dimerization of sortilin, we constructed expression vectors of FLAG-sortilin ECD plus transmembrane domain (TMD) and ICD+TMD and expressed them in HEK293 cells (Fig. 2*A*). In reducing Western blotting, protein expressions of FLAG-sortilin full length (Full), ECD+TMD, and ICD+TMD were detected as bands of plausible molecular size (Fig. 2*B*). In non-reducing Western blotting where disulfide bonds could be retained, FLAG-sortilin Full and ECD+TMD expressed a couple of bands (Fig. 2*C*). A band of 75–100 kDa was detected as monomers (Fig. 2*C*). Bands of ∼200 kDa and higher molecular mass were detected as homodimers and multimers with intermolecular disulfide bonds (Fig. 2*C*). Next, FLAG-sortilin Full and ECD+TMD were cross-linked using the water-soluble, non-cleavable cross-linker bis(sulfosuccinimidyl)suberate (BS^3^) in HEK293 cells. Both bands of homodimers and multimers appeared via the cross-linking (Fig. 2*D*). These data suggest that sortilin forms homodimers and multimers in the extracellular domain. Dimerization of FLAG-sortilin ICD+TMD was not clearly detected in the non-reducing Western blotting using whole-cell lysate, potentially due to low protein expression levels of FLAG-sortilin ICD+TMD (Fig. 2, *B* and *C*). Also, protein expression of FLAG-sortilin ICD+TMD was lower in HEK293 stably expressing FLAG-sortilin ICD+TMD (FLAG-sortilin ICD+TMD HEK293 cells). Therefore, we investigated whether FLAG-sortilin ICD+TMD would undergo degradation in proteasomes and lysosomes by adding a proteasome inhibitor, MG-132, and a lysosome inhibitor, chloroquine, to FLAG-sortilin ICD+TMD HEK293 cells, respectively. MG-132 increased protein expression of FLAG-sortilin ICD+TMD in a time- and concentration-dependent manner, whereas chloroquine did not, suggesting that FLAG-sortilin ICD+TMD is degraded in proteasomes (Fig. 2, *E* and *F*). To detect homodimers of FLAG-sortilin ICD+TMD, we performed incubation with MG-132 and immunoprecipitation with anti-FLAG antibody. In non-reducing Western blotting, bands with molecular size approximately twice as high as monomers of FLAG-sortilin ICD+TMD were detected (Fig. 2*G*). These data indicate that sortilin forms homodimers with an intermolecular disulfide bond in the intracellular domain.

### The transmembrane domain of sortilin forms homodimers via noncovalent interactions

**Figure 3.**
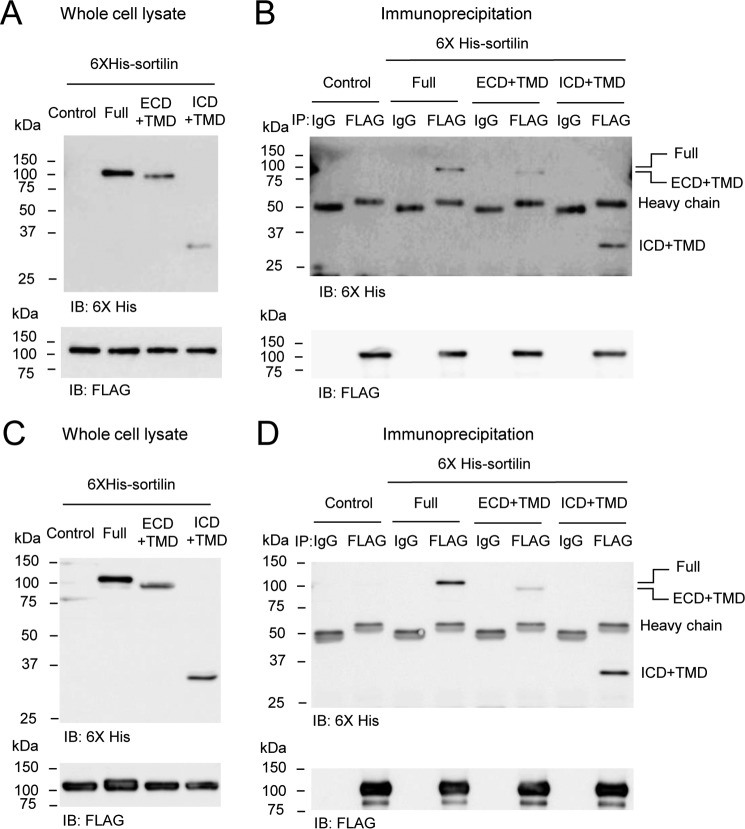
**The transmembrane domain of sortilin forms homodimers via noncovalent interaction.** *A–D*, His_6_-sortilin Full, ECD+TMD, and ICD+TMD were transiently overexpressed in HEK293 cells with stably overexpressed FLAG-sortilin Full (*A* and *B*) and ECD+TMD (*C* and *D*), respectively. Immunoprecipitation with anti-FLAG M2 antibody was performed using the cell lysates. Western blotting was carried out using whole-cell lysates (*A* and *C*) and immunoprecipitants (*B* and *D*). His_6_-sortilin Full, ECD+TMD, and ICD+TMD were coprecipitated with FLAG-sortilin Full or ECD+TMD (*B* and *D*) (*n* = 3). *IB*, immunoblotting.

To confirm dimerization of sortilin in ECD and ICD, we performed immunoprecipitation in HEK293 cells stably overexpressing FLAG-sortilin Full (FLAG-sortilin Full HEK293 cells or FLAG-sortilin HEK293 cells) where His_6_-sortilin Full, ECD+TMD, or ICD+TMD were transiently overexpressed, respectively (Fig. 3, *A* and *B*). His_6_-sortilin Full, ECD+TMD, and ICD+TMD were precipitated with FLAG-sortilin Full (Fig. 3*B*). Because their constructs have a transmembrane domain, the possibility that the transmembrane domain forms dimers remained. Therefore, to investigate dimerization of the transmembrane domain, we carried out immunoprecipitation using HEK293 cells stably overexpressing FLAG-sortilin ECD+TMD (FLAG-sortilin ECD+TMD HEK293 cells) where His_6_-sortilin ICD+TMD was transiently overexpressed (Fig. 3, *C* and *D*). His_6_-sortilin Full and ECD+TMD were also overexpressed as a positive control (Fig. 3, *C* and *D*). His_6_-sortilin Full, ECD+TMD, and ICD+TMD were precipitated with FLAG-sortilin ECD+TMD (Fig. 3*D*). Binding of FLAG-sortilin ECD+TMD and His_6_-sortilin ICD+TMD indicates that sortilin can form homodimers via noncovalent interaction in the transmembrane domain because FLAG-sortilin ECD+TMD and His_6_-sortilin ICD+TMD cannot bind together via their ECD and ICD.

### Substituting the transmembrane domain of sortilin with the corresponding domain of CD43 does not decrease the dimeric form of sortilin

**Figure 4.**
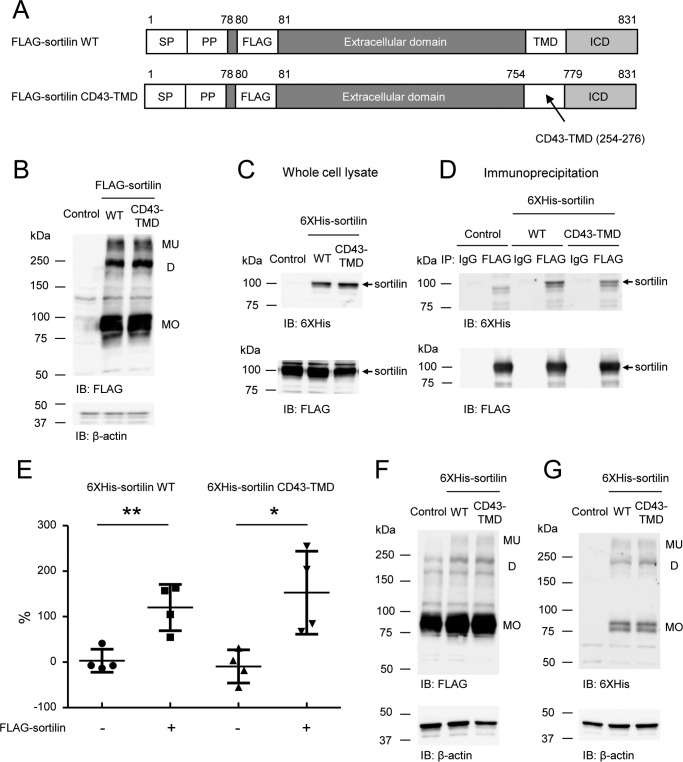
**Substituting the transmembrane domain of sortilin with the corresponding domain of CD43 does not decrease the dimeric form of sortilin.** *A*, schematic of FLAG-sortilin wildtype (WT) and FLAG-sortilin CD43-TMD. The transmembrane domain of sortilin was replaced with that of CD43. *SP*, signal peptide; *PP*, propeptide. *B*, FLAG-sortilin WT and FLAG-sortilin CD43-TMD were transiently overexpressed in HEK293 cells, and non-reducing Western blotting was carried out using cell lysate with anti-FLAG antibody (*n* = 3). Monomers, homodimers, and multimers are abbreviated as *MO*, *D*, and *MU*, respectively. *C* and *D*, His_6_-sortilin WT or His_6_-sortilin CD43-TMD was transiently overexpressed in HEK293 cells stably overexpressing FLAG-sortilin, and immunoprecipitation was performed using anti-FLAG M2 antibody. Western blotting was carried out using whole-cell lysates (*C*) and immunoprecipitants (*D*). His_6_-sortilin CD43-TMD coprecipitated with FLAG-sortilin as well as His_6_-sortilin WT. *Arrows*, sortilin wildtype or sortilin CD43-TMD (*n* = 3). *E*, in FLAG-sortilin HEK293 cells or HEK293 cells, His_6_-sortilin CD43-TMD was overexpressed. The cells were subjected to TR-FRET assay. Change of FRET signal by expression of His_6_-sortilin WT or CD43-TMD is indicated by percent change (mean ± S.D., *n* = 4, one independent experiment). *Error bars* represent S.D. *, *p* < 0.05; **, *p* < 0.01 by *t* test. *F* and *G*, in FLAG-sortilin HEK293 cells, His_6_-sortilin WT or His_6_-sortilin CD43-TMD were overexpressed. The cell lysates were subjected to non-reducing Western blotting with anti-FLAG antibody (*F*) and anti-His_6_ antibody (*G*), demonstrating that substituting the transmembrane domain of sortilin with that of CD43 did not decrease dimerization (*n* = 3). *IB*, immunoblotting.

To investigate the contribution of the transmembrane domain of sortilin to dimerization, the transmembrane domain was replaced with that of CD43, which does not form homodimers (sortilin CD43-TMD) as was reported previously (26) (Fig. 4*A*). Sortilin CD43-TMD formed homodimers in the non-reducing Western blotting (Fig. 4*B*). Also, His_6_-sortilin CD43-TMD was precipitated with FLAG-sortilin wildtype in the immunoprecipitation of HEK293 cells as well as His_6_-sortilin wildtype (Fig. 4, *C* and *D*), and coexpression of FLAG-sortilin and His_6_-sortilin CD43-TMD increased the FRET signal in HEK293 cells as well as that of FLAG-sortilin and His_6_-sortilin wildtype (Fig. 4*E*). Also, coexpression of His_6_-sortilin CD43-TMD and FLAG-sortilin increased dimerized FLAG-sortilin when compared with the control as well as that of His_6_-sortilin wildtype and FLAG-sortilin in the non-reducing Western blotting (Fig. 4*F*). In addition, His_6_-sortilin CD43-TMD formed homodimers at the same level as His_6_-sortilin wildtype (Fig. 4*G*). These data suggest that inhibiting dimerization of the transmembrane domain is not sufficient to suppress sortilin dimerization, possibly due to dimerization of the intracellular and extracellular domains via the covalent binding of intermolecular disulfide bonds.

### Mutation of Cys^783^ abolished dimerization of sortilin

**Figure 5.**
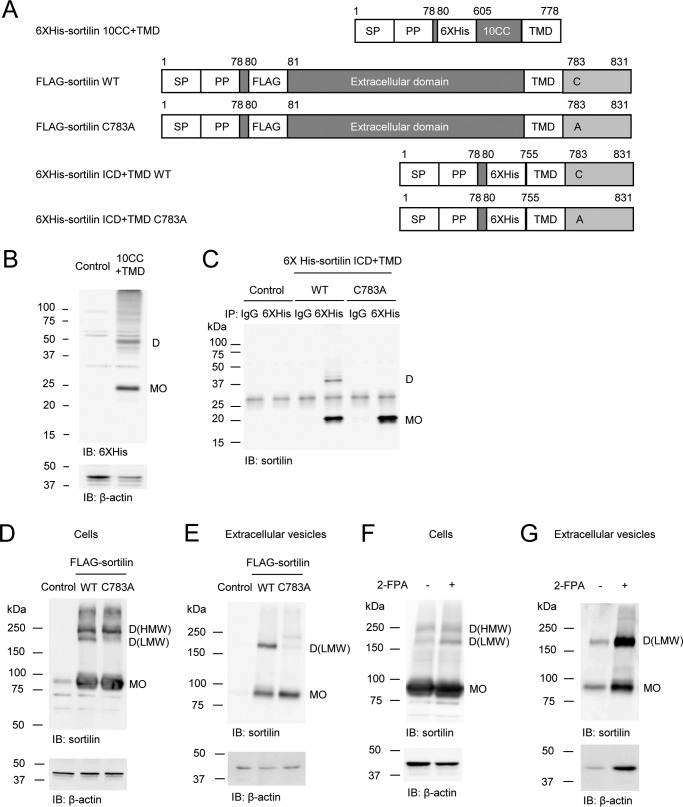
**Mutation of Cys^783^ abolishes dimerization of sortilin.** *A*, schematic of His_6_-sortilin 10CC+TMD, FLAG-sortilin WT and C783A, and His_6_-sortilin ICD+TMD WT and C783A. Cysteine 783 was replaced by alanine. *SP*, signal peptide; *PP*, propeptide. *B*, expression vector of His_6_-sortilin 10CC+TMD was transfected in HEK293 cells. Dimerization of His_6_-sortilin 10CC+TMD was detected in non-reducing Western blotting with anti-His_6_ antibody (*n* = 3). *C*, sortilin ICD+TMD C783A did not form homodimers in HEK293 cells in the non-reducing Western blotting (*n* = 3). *D* and *E*, C783A decreased sortilin homodimers of low molecular weight in the cells (*D*) and extracellular vesicles (*E*) of HEK293 cells in non-reducing Western blotting (*n* = 3). *F* and *G*, 24-h incubation with 2-FPA, an inhibitor of palmitoylation, increased sortilin homodimers of low molecular weight in HEK293 cells stably overexpressing FLAG-sortilin (*F*) and their extracellular vesicles (*G*) (*n* = 3). Monomers and homodimers of high and low molecular weight are abbreviated as *MO*, *D(HMW)*, and *D(LMW)*, respectively. *IB*, immunoblotting.

Previous reports have shown that cysteines play an important role in maintaining the structure of sortilin because they form intramolecular disulfide bonds in the extracellular domain of sortilin (28). In addition, in this study, we demonstrate that intermolecular disulfide bonds are formed within homodimers. Although the cysteines responsible for the formation of intermolecular disulfide bonds in the extracellular domain have not yet been identified, the 10CC (ten conserved cysteines) domain formed dimers in the non-reducing Western blotting (Fig. 5*B*), indicating that intermolecular disulfide bonds could be formed within the 10CC domain. Next, we investigated the intermolecular disulfide bond in the intracellular domain. Because sortilin has only one cysteine (Cys^783^) in the intracellular domain, the cysteine was expected to form an intermolecular disulfide bond for dimerization in the intracellular domain. When Cys^783^ was replaced by alanine (C783A) in His_6_-sortilin ICD+TMD and FLAG-sortilin Full (Fig. 5*A*), His_6_-sortilin ICD+TMD C783A did not form homodimers in HEK293 cells (Fig. 5*C*). Of note, FLAG-sortilin C783A decreased only homodimers of low molecular weight in HEK293 cells; it did not change those of high molecular weight and multimers (Fig. 5*D*). The homodimers of low molecular weight were mainly transported to EVs, whereas homodimers of high molecular weight and multimers were not transported to EVs (Fig. 5*E*). Therefore, FLAG-sortilin C783A significantly decreased transport of dimerized sortilin to EVs (Fig. 5*E*). Because Cys^783^ was reported to be palmitoylated (23), we examined the connection between palmitoylation and intermolecular disulfide bond by incubating FLAG-sortilin HEK293 cells with 2-fluoropalmitic acid (2-FPA), an inhibitor of palmitoylation (23), which increased only homodimers of lower molecular size in the cells (Fig. 5*F*). Our data indicate that the Cys^783^ residue is shared by two processes, palmitoylation and the formation of intermolecular disulfide bond. Also, 2-FPA increased dimerization of FLAG-sortilin in the EVs (Fig. 5*G*), suggesting that the decrease of the homodimers in the EVs via the mutation of Cys^783^ is due to a decrease in dimerization but not palmitoylation (Fig. 5*E*).

### Binding of sortilin-derived propeptide suppresses dimerization of sortilin

**Figure 6.**
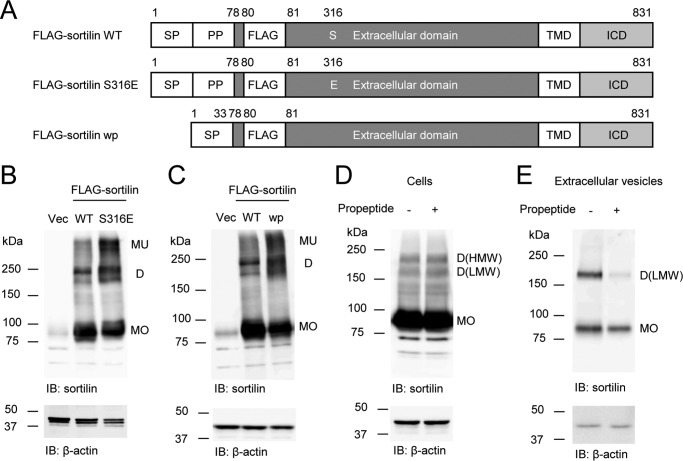
**Sortilin S316E and sortilin wp increase dimerization in HEK293 cells, and the addition of propeptide decreases dimerization in the extracellular vesicles of FLAG-sortilin HEK293 cells.** *A*, schematic of FLAG-sortilin WT, S316E, and wp. Serine 316 was replaced by glutamic acid in FLAG-sortilin S316E. Propeptide was removed in FLAG-sortilin wp. *SP*, signal peptide; *PP*, propeptide. *B*, S316E increased dimerization of sortilin in HEK293 cells (*n* = 3). *C*, removal of propeptide increased dimerization of sortilin in HEK293 cells (*n* = 3). *D* and *E*, addition of propeptide (100 nmol/liter) decreased dimerization of sortilin in the extracellular vesicles of FLAG-sortilin HEK293 cells (*E*), whereas a decrease in the cells was not observed (*D*) (*n* = 2). Monomers and homodimers of high and low molecular weight are abbreviated as *MO*, *D(HMW)*, and *D(LMW)*, respectively. *Vec*, vector; *IB*, immunoblotting.

The structure of the Vps10p domain in both sortilin and SorLA has been reported (29, 30). SorLA has a different configuration in a ligand-free state or propeptide-bound state (29). Similar changes may take place in sortilin, and these different states may form monomers and homodimers of sortilin. Because the S316E mutation inhibits binding with sortilin-derived propeptide (30), we used S316E mutant (Fig. 6*A*) to investigate the effects of the propeptide binding on dimerization. S316E increased dimerization in HEK293 cells concomitantly with a decrease in monomers (Fig. 6*B*). To further validate the effect of the propeptide binding on dimerization, sortilin without propeptide (wp) (28) was constructed (Fig. 6*A*) and overexpressed in HEK293 cells. Sortilin wp also increased the dimerization in HEK293 cells concomitantly with a decrease in monomers (Fig. 6*C*). Also, the addition of sortilin-derived propeptide decreased dimerization in the EVs of FLAG-sortilin HEK293 cells (Fig. 6*E*), although it did not affect dimerization in the cells (Fig. 6*D*).

### Soluble sortilin forms homodimers

**Figure 7.**
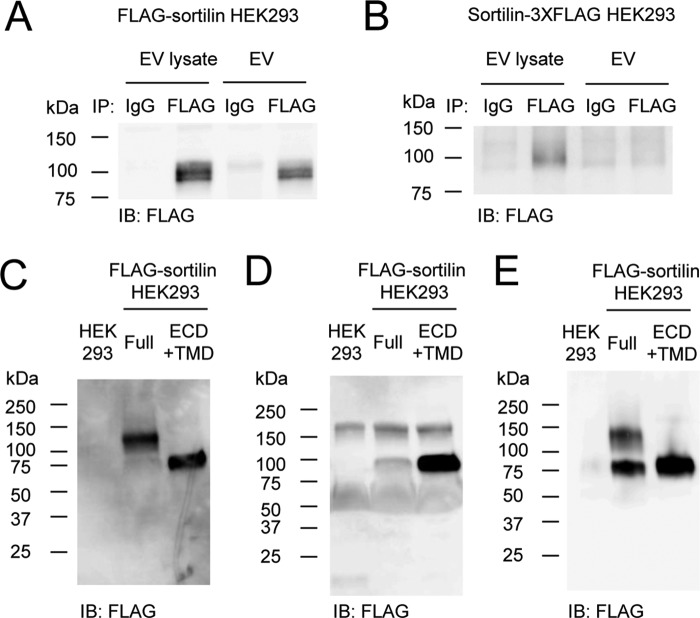
**Soluble sortilin forms homodimers.** *A* and *B*, orientation of sortilin on the EV membrane was determined using EVs secreted from FLAG-sortilin HEK293 cells (*A*) and sortilin-3XFLAG HEK293 cells (*B*). EVs or their lysates were subjected to immunoprecipitation with anti-FLAG M2 antibody, and FLAG-sortilin (*A*) or sortilin-3XFLAG (*B*) was detected by Western blotting with anti-FLAG antibody, showing that the extracellular domain of sortilin is located outside of EVs (*n* = 3). *C* and *D*, soluble sortilin secreted by HEK293 cells overexpressing FLAG-sortilin Full and FLAG-sortilin ECD+TMD was detected in non-reducing (*C*) and reducing Western blotting (*D*), showing that they were homodimers and monomers, respectively (*n* = 3). *E*, soluble sortilin secreted by HEK293 cells overexpressing FLAG-sortilin Full and FLAG-sortilin ECD+TMD was purified and detected in non-reducing Western blotting. *IB*, immunoblotting.

Previous studies reported that serum sortilin levels are associated with cardiovascular risk, such as aortic calcification (31) and atherothrombosis (32), as well as depression (33). Soluble sortilin can also activate survival pathways in cancer cells (6, 34). Therefore, it is important to understand whether soluble sortilin contributes to diseases through the formation of monomers and/or homodimers. In addition, it is critical to determine the orientation of sortilin on the EV membrane for the detection of soluble sortilin and sortilin within EVs. Because serum sortilin levels have been measured using antibodies against the extracellular domain of sortilin, these antibodies could detect both forms of sortilin as long as the extracellular domain of sortilin is located outside of EVs. Therefore, to determine the orientation of sortilin on the EV membrane, we performed an immunoprecipitation assay using EVs secreted from FLAG-sortilin Full HEK293 cells and HEK293 cells stably expressing sortilin with 3XFLAG at the C terminus (sortilin-3XFLAG). FLAG-sortilin was detected in EVs and the lysate (Fig. 7*A*), but sortilin-3XFLAG was detected in the lysate only (Fig. 7*B*), suggesting that the extracellular domain of sortilin is located outside of EVs.

To investigate whether soluble sortilin forms homodimers or monomers, we performed non-reducing Western blotting using EV-deprived culture medium of FLAG-sortilin HEK293 cells. The molecular size of soluble sortilin was calculated as ∼120 kDa in the non-reducing Western blotting (Fig. 7*C*). Because this size is higher than that detected in the reducing Western blotting (Fig. 7*D*), the band of 120 kDa was detected as homodimers. Forms of soluble sortilin secreted from FLAG-sortilin ECD+TMD HEK293 cells were also examined because the intracellular domain of sortilin can be cleaved (7, 35). Soluble sortilin from FLAG-sortilin ECD+TMD HEK293 cells was detected as a band at ∼80 kDa (Fig. 7*C*) in the form of monomers. Soluble sortilin was then purified using an anti-FLAG antibody affinity column using EV-deprived culture medium. Soluble sortilin secreted from FLAG-sortilin HEK293 cells was detected as bands of 80 and 120 kDa, which associate with dimers of soluble sortilin that partially changed their form to monomers during the process of purification (Fig. 7*E*). The formation of the band at 120 kDa indicates that the soluble sortilin exists in the form of homodimers.

## Discussion

We previously demonstrated that sortilin promotes vascular calcification via its trafficking of tissue-nonspecific alkaline phosphatase, a facilitator of calcification, to EVs (20). Other groups also reported that sortilin promotes exosome release and forms a complex with two tyrosine receptors, tropomyosin-related kinase B (TrkB) and epidermal growth factor receptor, which play an important role in the control of the cancer cell microenvironment and tumor angiogenesis (7). Given these results, the major objective of our study was to understand how sortilin is transported to EVs to potentially inhibit the atypically high expression levels observed in multiple diseases, including cardiovascular disease (9, 20). We specifically overexpressed tagged sortilin to obtain more definitive results for the dimerization of sortilin, taking into consideration the higher expression levels of sortilin in pathologic conditions.

**Figure 8.**
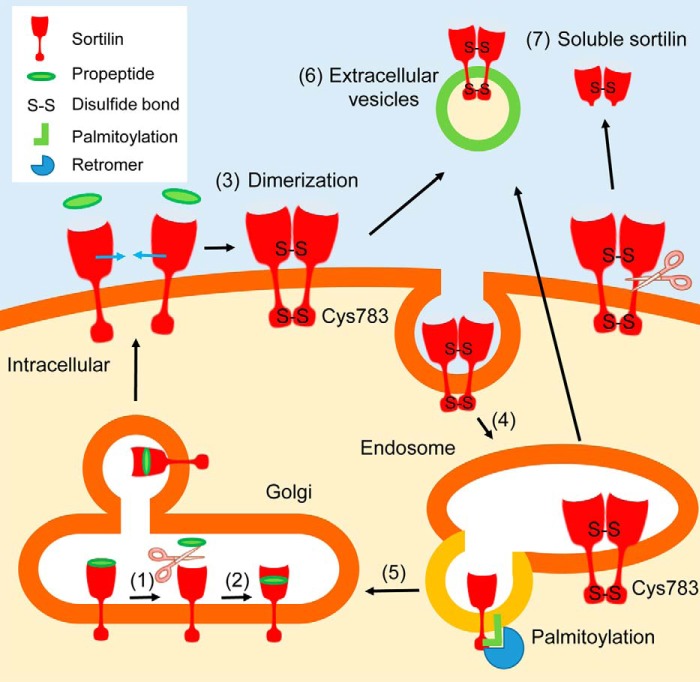
**Schematic showing involvement of dimerization for trafficking of sortilin.** *1*, propeptide is cleaved from sortilin. *2*, propeptide binds to sortilin at a different location. Then sortilin is transported through the Golgi apparatus. *3*, sortilin forms homodimers with intermolecular disulfide bonds at the 10CC domain and/or Cys^783^ possibly in the absence of propeptide. *4*, sortilin is incorporated into the endosome by endocytosis. *5*, palmitoylated sortilin is transported back to the Golgi apparatus via its interaction with retromers. *6*, sortilin homodimer is secreted by extracellular vesicles (microvesicles and/or exosomes). *7*, sortilin homodimer is shed and secreted as soluble sortilin.

We demonstrated that, in the intracellular domain of sortilin, Cys^783^ forms an intermolecular disulfide bond to generate homodimers. Because Cys^783^ has been reported to be palmitoylated (23), formation of an intermolecular disulfide bond could compete with palmitoylation at Cys^783^. To confirm this, we demonstrated that a palmitoylation inhibitor increased sortilin dimerization. Importantly, we showed that dimerized sortilin with an intermolecular disulfide bond at Cys^783^ acts as the main dimer transported to EVs, and the transport of dimerized sortilin to EVs ceases when the intermolecular disulfide bond at Cys^783^ is lost via mutation. Because the palmitoylation inhibitor increased transport of dimerized sortilin to EVs, formation of an intermolecular disulfide bond at Cys^783^ residue could facilitate transport of dimerized sortilin to EVs. This could be due to the fact that palmitoylation accelerates sortilin trafficking to the Golgi apparatus through interaction with retromers (23), which recognize cargo proteins, such as mannose 6-phosphate receptor and sortilin, and retrieve them from the endosome to the Golgi apparatus (36–40) (Fig. 8). Our future studies will address whether sortilin that is transported from the endoplasmic reticulum to the Golgi apparatus is also regulated via palmitoylation and dimerization.

Our immunoprecipitation experiments showed that, in the transmembrane domain of sortilin, a noncovalent interaction occurs to form homodimers. Inhibiting binding in the transmembrane domain, however, was not sufficient to suppress dimerization of sortilin. This could be explained by sortilin covalent binding in the intracellular and extracellular domains while concurrently other type I transmembrane proteins, such as PSGL-1 (26) and amyloid precursor protein (41), form homodimers through the transmembrane domain as reported previously.

Previous reports have suggested that, in the extracellular domain, the 10CC domain exhibited intramolecular disulfide bonds formed by 10 cysteines (28). However, our results support the possibility that some cysteines in the 10CC domain contribute to the formation of intermolecular disulfide bonds for homodimers. Because C783A mutant decreased only homodimers of low molecular weight, homodimers with cysteines in the 10CC domain could be formed, and the C783A mutant could exist as homodimers of high molecular weight and multimers. Based on the previous hypothesis that interaction of the propeptide-binding site and 10CC domain occurs (30), we posited that binding of sortilin-derived propeptide affects dimerization. Because the structure of SorLA, which, like sortilin, has a Vps10p domain, can be changed in a ligand-free state or propeptide-bound state (29), we proposed that these two different states contribute to the formation of either monomers or homodimers of sortilin (Fig. 8). We further showed that both sortilin S316E and sortilin without propeptide exhibit increased homodimer formation, whereas the addition of propeptide reduced homodimer formation. These data strongly suggest that sortilin dimerization is controlled through ligand binding and subsequent conformational change of the Vps10p domain, especially the 10CC domain. As the ligand regulated dimerization of sortilin, our future studies will investigate the effects of other ligands, such as progranulin (16) and neurotensin (42), in addition to sortilin-derived propeptide on sortilin dimerization.

The present study demonstrates that soluble sortilin exists as homodimers. Moreover, the intracellular domain is essential for the dimerization of soluble sortilin. Previous studies using sortilin overexpressed as recombinant protein lacking both the transmembrane and intracellular domains demonstrate the presence of soluble sortilin in the form of monomers (34, 43). Our study demonstrates the method to produce dimerized soluble sortilin. In the purification process of dimerized soluble sortilin, some intermolecular disulfide bonds could be destroyed, which would result in monomer formation; thus, the purification procedure needs to be improved.

Our findings of dimerized soluble sortilin have important implications in a clinical setting because serum sortilin can act as a biomarker for cardiovascular and neurologic diseases (31–33). Therefore, it is important to clarify the differences between monomers and dimers of soluble sortilin and the monomer/dimer ratio in serum for these diseases. This would allow for a more accurate diagnosis for the diseases, similar to the detection of high molecular weight adiponectin for metabolic syndrome (44, 45). We also determined that the extracellular domain of sortilin is located outside of EVs. This finding validates the possibility that the detection of sortilin-positive EVs is possible using antibodies against the extracellular domain. In the future, clarifying the association of sortilin-containing EVs with various diseases would be useful as a clinically relevant surrogate for disease progression. In fact, the prospective for using exosomal proteins in disease diagnosis and prognosis prediction has been increasing (46).

In conclusion, we demonstrated that sortilin forms homodimers, which likely play an important role in the trafficking of sortilin to the EVs and may be regulated using intermolecular disulfide bonds. In addition, our data suggest that sortilin-derived propeptide controls the dimerization of sortilin and therefore the possibility of its regulation via ligand binding in the extracellular domain. Based on our findings, we propose that, in the future, molecules inhibiting sortilin dimerization, such as small-molecule compounds, antibodies, and peptides, will provide new therapeutic means to treat EV-associated diseases, including vascular calcification and cancer, by suppressing transport of sortilin and disease-causing proteins bound with sortilin to EVs.

## Experimental procedures

### Chemicals and reagents

MG-132 (catalog number M7449) and chloroquine diphosphate salt (catalog number C6628) were purchased from Sigma-Aldrich. 2-FPA (catalog number 90380) was purchased from Cayman Chemical. Human sortilin propeptide (sortilin-derived propeptide) (catalog number 049-75, lot number 431019) was purchased from Phoenix Pharmaceuticals, Inc. Primers were purchased from Integrated DNA Technologies, Inc. PCR reagents were purchased from EMD Millipore Corp.

### Vectors and constructs

Expression vectors were constructed in pcDNA3.1(+) vector (Thermo Fisher Scientific Inc., catalog number V79020). The following constructs of human sortilin (NM_002959) were generated by inserting FLAG (DYKDDDDK) or His_6_ (HHHHHH) tag into 3 amino acids (^78^SAP^80^) behind the furin cleavage site ^74^RWRR^77^ (42) using site-directed mutagenesis: pcDNA3.1(+) FLAG-sortilin Full, amino acids (aa) 1–831; pcDNA3.1(+) FLAG-sortilin ECD+TMD, aa 1–778; pcDNA3.1(+) FLAG-sortilin ICD+TMD, aa 1–831 (Δ81–754); pcDNA3.1(+) His_6_-sortilin full length (Full); pcDNA3.1(+) His_6_-sortilin ECD+TMD; pcDNA3.1(+) His_6_-sortilin ICD+TMD; pcDNA3.1(+) His_6_-sortilin 10CC domain+TMD, aa 1-778 (Δ81–604); pcDNA3.1(+) FLAG-sortilin C783A; pcDNA3.1(+) His_6_-sortilin ICD+TMD C783A; pcDNA3.1(+) FLAG-sortilin S316E; pcDNA3.1(+) FLAG-sortilin wp, aa 1–831 (Δ34–77). The following constructs of sortilin CD43-TMD were generated by an overlapping PCR strategy using a CD43 expression vector (Origene, catalog number RC204195, NM_003123) (26): pcDNA3.1(+) FLAG-sortilin CD43-TMD, sortilin aa 1–831 (Δ755–778) with CD43 aa 254–276; pcDNA3.1(+) His_6_-sortilin CD43-TMD. The expression vector of sortilin with 3XFLAG tag at the C terminus (sortilin-3XFLAG; catalog number EX-M0397-M14) was purchased from GeneCopoeia, Inc.

### Western blot analysis

Cells, EVs, and the supernatant of culture medium were lysed with IP lysis buffer (Thermo Fisher Scientific Inc., catalog number 87787) containing protease inhibitor (Roche Diagnostics, catalog number 04693159001). Protein concentration was measured using the bicinchoninic acid (BCA) method (Thermo Fisher Scientific Inc., catalog number 23225). Laemmli buffer (Boston Bioproduct; non-reducing, catalog number BP-110NR; reducing, catalog number BP-111R) was added to the lysate and boiled at 95 °C for 5 min. Total protein was separated by SDS-PAGE and transferred to polyvinylidene difluoride (PVDF) or nitrocellulose membrane using the iBlot Western blotting system (Life Technologies) or conventional wet method. Primary antibodies against human sortilin ICD (rabbit, 1:1000; Abcam plc, catalog number ab16640, lot number GR185198-1), human β-actin (mouse, 1:2000; Novus Biologicals, LLC, catalog number NB600-501, lot number 014M4759), FLAG (rabbit, 1:1000; Sigma, catalog number F4725, lot number 093M4798), His_6_ (mouse, 1:1000; Abcam plc, catalog number ab18184, lot number GR247674-1) were used.

### Immunoprecipitation

Cells or EVs were lysed in IP lysis buffer. Anti-FLAG M2 antibody (5 μg; Sigma, catalog number F1804, lot number SLBJ4607V) or mouse IgG (5 μg; R&D Systems, catalog number MAB002, lot number IX2415091) was incubated with Dynabeads with Protein G (Thermo Fisher Scientific Inc., catalog number 10004D) by rotation overnight at 4 °C. Cell lysates, EVs, or their lysates were incubated with the beads bound to anti-FLAG M2 antibody or mouse IgG for 4 h at 4 °C under rotating conditions. The bead-antibody-protein complex was washed with PBS three times. Then Laemmli buffer was added to the precipitates for SDS-PAGE.

### Cross-linking experiment

Chemical cross-linking was carried out by incubating HEK293 cells transiently overexpressing FLAG-sortilin Full or ECD+TMD with 1 mmol/liter BS^3^, a water-soluble, non-cleavable cross-linker (Thermo Fisher Scientific Inc., catalog number 21580), at room temperature for 30 min according to the manufacturer's protocol and previous reports (47, 48) with slight modification. The reaction was stopped with 15-min incubation of 10 m mol/liter Tris-HCl, pH 7.4, and cells were centrifuged at 1,000 rpm for 5 min to remove the buffer, including BS^3^, and washed with PBS. Then cells were lysed in IP lysis buffer for Western blotting.

### Cell culture of HEK293 cells and establishment of transfectants

HEK293 cells were purchased from American Type Culture Collection (ATCC) and maintained in Eagle's minimum essential medium (ATCC, catalog number 30-2003) supplemented with 10% fetal bovine serum (FBS), 100 units/ml penicillin, and 100 μg/ml streptomycin at 37 °C in a humidified atmosphere of 5% CO_2_. For transfection in HEK293 cells, Lipofectamine 2000 reagent (Thermo Fisher Scientific Inc., catalog number 11668019) was used according to the manufacturer's protocol. HEK293 cells stably expressing FLAG-sortilin Full, ECD+TMD, ICD+TMD, and sortilin-3XFLAG were obtained by transfection with pcDNA3.1(+) FLAG-sortilin Full, pcDNA3.1(+) FLAG-sortilin-ECD+TMD, pcDNA3.1(+) FLAG-sortilin ICD+TMD, and expression vector of sortilin-3XFLAG (GeneCopoeia, Inc., catalog number EX-M0397-M14), respectively. These cell lines were maintained in Eagle's minimum essential medium supplemented with 10% FBS, 100 units/ml penicillin, 100 μg/ml streptomycin, and 800 μg/ml geneticin. Cells were incubated in an incubator with MG-132 and chloroquine for the indicated times and with 2-FPA and sortilin propeptide for 24 h.

### Separation of culture medium to supernatant and EVs

Separation of culture medium into supernatant and EVs was performed according to the protocol reported by our group (49). Culture medium underwent centrifugation at 1,000 rpm for 5 min to remove cell debris. Then the supernatant and EVs were separated by ultracentrifugation at 100,000 × *g* for 40 min at 4 °C (Optima Max Ultracentrifuge, Beckman Coulter).

### TR-FRET and HTRF

TR-FRET and HTRF were performed as described (27). FLAG-sortilin Full HEK293 cells were harvested using cell dissociation solution (Sigma, catalog number C5914) 24 h after transfection of His_6_-sortilin expression vector. Incubation on a circular rotator was performed at 4 °C with 1 × 10^6^ cells/ml for TR-FRET and 2 × 10^6^ cells/ml for HTRF containing 1 nmol/liter anti-FLAG (M2)-cryptate (Cisbio Bioassays, catalog number 61FG2KLA, lot number 25A) and 3 nmol/liter anti-6HIS-XL665 (Cisbio Bioassays, catalog number 61HISXLA, lot number 56A) in PBS supplemented with 25% FBS. For TR-FRET, cells were centrifuged at 1,000 rpm for 5 min to remove the antibodies, resuspended in PBS, and applied into a 96-well white plate. For HTRF, cells were applied without removing the antibodies into a 96-well white plate. Then the plate was read (excitation, 320 nm; emission, 620 nm (cutoff, 570 nm), 665 nm (cutoff, 630 nm); delay, 50 μs; integration, 500 μs). The FRET signal was calculated as the (Ratio of counts/s 665:620) × 10,000, and percent change of the FRET signal by His_6_-sortilin expression was expressed.

### Purification of soluble sortilin

EV-deprived culture medium of FLAG-sortilin Full or ECD+TMD HEK293 cells was subjected to anti-FLAG M2 Affinity Gel (Sigma, catalog number A2220). Soluble sortilin with FLAG tag was eluted with 100 μg/ml FLAG peptide (Sigma, F3290, lot number SLBR6767V). Purified soluble sortilin was dialyzed in dialysis buffer (50 mmol/liter Tris-HCl, 150 mmol/liter NaCl, pH 7.4).

### Statistical analysis

Data are presented as means ± S.E. of the indicated number. A Student's *t* test was used to determine the significance of differences in comparisons. Values of *p* < 0.05 were considered statistically significant.

## Author contributions

S. I. and E. A. conceptualization; S. I. data curation; S. I. formal analysis; S. I. validation; S. I. investigation; S. I. methodology; S. I. writing-original draft; K. M. and E. A. project administration; K. M., M. A., and E. A. writing-review and editing; M. A. funding acquisition; E. A. supervision.

## Footnotes

3 The abbreviations used are:

EV: extracellular vesicle
Full: full length
ECD: extracellular domain
ICD: intracellular domain
TMD: transmembrane domain
wp: without propeptide
2-FPA: 2-fluoropalmitic acid
aa: amino acids
BS^3^: bis(sulfosuccinimidyl)suberate
TR-FRET: time-resolved fluorescence energy transfer
HTRF: homogenous TR-FRET
aa: amino acids
IP: immunoprecipitation.
